# Perceived Quality of Life and Life Satisfaction: Does the Role of Gender, Age, Skills, and Psychological Factors Remain Relevant after the COVID-19 Pandemic?

**DOI:** 10.3390/children10091460

**Published:** 2023-08-27

**Authors:** Cátia Branquinho, Bárbara Moraes, Catarina Noronha, Tomás Ferreira, Nuno Neto Rodrigues, Margarida Gaspar de Matos

**Affiliations:** 1 Aventura Social Project, 1400-415 Lisbon, Portugal; catiasofiabranquinho@gmail.com (C.B.); barbaracsmoraes@hotmail.com (B.M.); catarinanoronha26@live.com.pt (C.N.); 2 Institute of Environmental Health, Medicine Faculty, University of Lisbon, 1649-026 Lisbon, Portugal; 3 Dream Teens/Aventura Social Project, 1400-415 Lisbon, Portugal; tomasmartimferreira4@gmail.com; 4 Directorate-General for Education and Science Statistics, 1399-054 Lisbon, Portugal; nuno.rodrigues@dgeec.medu.pt; 5 Faculty of Human Sciences, Portuguese Catholic University, 4169-005 Porto, Portugal; 6 Applied Psychology Research Center Capabilities & Inclusion, ISPA—University Institute, 1149-041 Lisbon, Portugal

**Keywords:** adolescents, psychological health and well-being, perceived quality of life and life satisfaction, gender, skills

## Abstract

Background: After two years of psychological, physical, social, economic, environmental, and societal challenges, this paper examines the psychological health and well-being of Portuguese students based on their socioemotional skills (SSES), positive youth development (PYD), depression, anxiety, and stress (DASS), as well as the relationship between these variables and their influence on perceived quality of life and life satisfaction. Methods: This study examined 3235 students from lower to upper secondary, half of whom were female (*M* = 14.46 ± 1.883 years old). Using SPSS software, descriptive statistics were determined for all variables; mean differences between age and gender were found using ANOVA and the post hoc Scheffe test. Linear regressions with the Enter method were used to study how to predict perceived quality of life and satisfaction with life. Results: Males had scores indicating more SSES|optimism, emotional control, resilience, confidence, sociability, creativity, energy, a sense of belonging to school, and PYD. Girls had better skills for cooperating and relating to teachers but more test anxiety and DASS. Younger adolescents had better psychological health, greater skills, and a better perception of quality of life and life satisfaction when compared to older adolescents. Age, gender, SSES, PYD, and the DASS variables can explain 69% of the variance in life satisfaction, while these variables can explain 60.5% of the variance in perceived quality of life. Conclusions: These results point to the relevance of SSES for psychological health and well-being, suggesting that interventions should focus on promoting these variables, paying special attention to female gender and age-related challenges.

## 1. Introduction

The pandemic had a particularly negative impact on adolescent age groups’ on physical and psychological health [1,2], but also on social [3,4] and academic levels [5,6]. Systematic literature reviews, coping reviews, and meta-analyses have reported a decline in physical activity [7]; increased symptoms of anxiety, depression, loneliness, stress, fear, tension, anger, fatigue, confusion, and worry [8]; social disconnection with the loss of important life moments [9]; global learning difficulties, initiated during the pandemic and sustained over time [10]. The pandemic context brought fewer opportunities for social interaction, which led to weaker social skills in children and adolescents [11,12,13], lower levels of empathy and pro-social behavior [14], feelings of loneliness, and poorer coping strategies and emotional self-regulation [15]. In tandem with this, a rise in bullying and cyberbullying was also observed [2,12].

Considering gender disparities, girls were the gender most impacted by the pandemic in terms of psychological health and well-being [16,17,18], exhibiting damage to self-esteem [19].

When examining the various socio-emotional competencies, girls demonstrated greater ability, particularly in social awareness, pro-social behavior, and responsible decision-making, whereas boys demonstrated greater self-management ability and motivation [20]. Taking age into account, this variable has an inverse relationship with socio-emotional skills, i.e., older youth demonstrate a progressive decline in socio-emotional skills such as self-esteem, connection to school [21], self-management, motivation, and decision-making [20].

From a positive developmental analysis perspective [22], the literature identifies higher levels of connectedness, character, and caring in girls and higher levels of competence and confidence in boys, who also exhibit a higher level of psychological adjustment [23,24,25]. According to Gómez-Baya et al. [25], girls tend to underestimate themselves more than boys do, and as a result, they have lower levels of self-esteem, self-concept, and well-being.

A positive development is associated with the efficacy of mental health problem prevention practices [26,27,28]. Psychological adjustment and psychological health are constructs that relate to the skills that subjects develop to cope with environmental challenges, thereby fostering a higher level of well-being, quality of life, and life satisfaction. In response to the challenges posed by the COVID-19 pandemic, young people’s skill deficits manifested as psychopathological symptoms. Depression, anxiety, and stress symptoms stand out among the primary symptoms due to their high prevalence [16,18,29].

Ravens-Sieberer et al. [26] observed the prevalence of psychosomatic symptoms (such as sleep problems), depressive symptoms, and anxious symptoms in a study conducted at the onset of the pandemic. In the longitudinal version of this study, the authors found a significant decline in the quality of life of participating children and adolescents, as well as an increase in the prevalence of emotional fragility and symptoms of anxiety and depression [30]. During this period, it was demonstrated that females are more susceptible to developing symptoms, and older adolescents report engaging in riskier behavior [27]. Anxiety, behavioral issues, and emotional vulnerability all increase with age [21].

The present study looks at the psychological health and well-being of Portuguese students based on their socio-emotional skills (SSES), positive youth development (PYD), depression, anxiety, and stress (DASS), as well as the relationship between these variables and their effects on perceived quality of life and life satisfaction.

**H1.**  *Gender differences exist for socio-emotional skills (SESS), positive youth development (PYD), depression, anxiety, and stress (DASS).*

**H2.**  *Socio-emotional skills (SESS), positive youth development (PYD), and depression, anxiety, and stress (DASS) vary with age.*

**H3.**  *There is a correlation between gender, age, socio-emotional skills (SESS), positive youth development (PYD), depression, anxiety, and stress.*

**H4.**  *Gender, age, socioemotional skills (SSES), positive youth development (PYD), depression, anxiety, and stress (DASS) are predictors of perceived quality of life.*

**H5.**  *Gender, age, socioemotional skills (SESS), positive youth development (PYD), depression, anxiety, and stress (DASS) all predict life satisfaction.*

With the answers to these hypotheses, we hope to contribute not only to the investigation of the current landscape of the psychological health and well-being of adolescents, which has been extensively studied, but also to the relationship between SSES, PYD, and DASS with perceived quality of life and life satisfaction, which we did not find to be extensively studied and which could be a key focus for the development or adaptation of health promotion interventions.

## 2. Materials and Methods

In the study “Psychological Health and Well-being|Observatory of Psychological Health and Well-being: Monitoring and Action”, the Directorate-General for Education and Science Statistics, the Directorate-General for Education, the National Program for the Promotion of School Success, the Aventura Social Team/ISAMB/University of Lisbon, the Order of Portuguese Psychologists, and the Calouste Gulbenkian Foundation collaborated and approved all the instruments used. This investigation began in December 2021 with Ministry of Education approval.

The sample consisted of a stratified (non-proportional) and random selection of Portuguese mainland public schools by geographic region/NUTS III (Nomenclature of Territorial Units for Statistical Purposes). In total, 27 groups of schools were included in which classes were also drawn, including all levels of education available in the school cluster (sample power estimation was 97%; error < 0.03). In February and March 2022, after stratified and random selection of each grade’s classes, the liaison teachers and psychologists of the participating schools administered the data collection instruments in computer rooms. Students only completed online questionnaires with parental informed consent. The application protocol lasted between 20 and 30 min on average. Methods and outcomes are described in detail in the online study report [31] that is accessible via the Internet.

### 2.1. Participants

This study included 3235 pupils aged 11 to 18 (*M* = 14.46 ± 1.883 years) from lower secondary (7th grade/±13 years old = 14.8%, 8th grade/±14 years old = 16.9%, 9th grade/±15 years old = 16.3%) to upper secondary (10th grade/±16 years old = 13.9%, 11th grade/±17 years old = 23%, 12th grade/±18 years old = 15.5%). In total, 46.1% of respondents were male, 50% were female, and 3.9% did not respond.

### 2.2. Measures

In the present study, the following variables were investigated:

- Social and Emotional Skills Questionnaire (SSES) [32], examined through the dimensions of optimism (8 items; α = 0.865), emotional control (8 items; α = 0.794), resilience/resistance (8 items; α = 0.883), confidence (8 items; α = 0.854), curiosity (8 items; α = 0.840), sociability (8 items; α = 0.827), persistence/perseverance (8 items; α = 0.857), creativity (8 items; α = 0.828), energy (8 items; α = 0.817), cooperation (8 items; α = 0.867), self-control (8 items; α = 0.842), sense of belonging to school (6 items; α = 0.721), and test anxiety (3 items; α = 0.84) (a five-point Likert scale, where 0 = strongly disagree and 4 = strongly agree); bullying (4 items; α = 0.756) and relationships with teachers (3 items; α = 0.808) (a four-point Likert scale, where 0 = never or rarely and 3 = once a week or more frequently). This instrument was used in a national study by the Calouste Gulbenkian Foundation, which included a Portuguese sub-sample [32].
- Positive Youth Development (PYD; [33], validated and adapted for the Portuguese population by Tomé et al. [34]) was assessed through the dimensions of competence (a five-point Likert-type scale where 0 = Strongly disagree and 4 = Strongly agree; 6 items; α = 0.823), confidence (a five-point Likert-type scale where 0 = Strongly disagree and 4 = Strongly agree; 6 items; α = 0.904), and connection (a five-point Likert-type scale where 0 = Strongly disagree and 4 = Strongly agree; except for two items where 0 = never true to 4 = always true; 8 items; α = 0.849).
- Depression, Anxiety, and Stress (DASS; [35], validated and adapted for the Portuguese population by Pais-Ribeiro et al. [36]) and for pre-adolescents and adolescents by Leal et al. [37]), based on the study of depression (7 items; α = 0.898), anxiety (7 items; α = 0.861), stress (7 items; α = 0.892) (a four-point Likert scale, where 0 = does not apply to me at all and 3 = applies to me most of the time) to analyze the data.
- Perceived Quality of Life [38] was assessed using five items on a six-point Likert scale (α = 0.863), with 0 = never and 5 = all time.
- Life Satisfaction [39] was measured through the item “The top of the ladder is …10 and represents the best possible life for you, the bottom of the ladder is “0” and represents the worst possible life for you. Right now, where do you think you stand on the ladder?”.

### 2.3. Data Analysis

→ Using SPSS version 25.0 for quantitative analysis, the data were analyzed. After testing the assumptions of normality with the Kolmogorov–Sminorv test for gender and school year (guaranteed except for SES|Emotional control, invoking the Central Limit Theory), and homogeneity, which was not guaranteed in the majority of the variables, but was prioritized due to the similar number of cases in each subgroup and the robustness of the parametric tests in the face of violation.
→ Starting with descriptive statistics (mean, standard deviation, minimum, and maximum) for all variables under consideration, ANOVA tests were used to look at differences in means based on gender and level of education. The post hoc Scheffe test was used to control multiple comparisons whenever there were more than two conditions. This test was prioritized since it was intended to investigate all possible contrasts between the means of the groups and can be used when there is or is not an equality of cases in the groups. Using the Pearson test, a cross-sectional analysis was conducted to confirm the relationship between the variables under investigation and to ascertain their magnitude and effect. The correlational analysis and literature review bolstered the need for an in-depth examination of the variables gender, age, SSES, PYD, and DASS in predicting perceived quality of life and life satisfaction.
→ Linear regressions were performed with the Enter method to assess the prediction of perceived quality of life and life satisfaction. The assumptions of the model, namely normal distribution, homogeneity, and error independence, were analyzed. The first two hypotheses were validated graphically, while the independence hypothesis was validated using the Durbin–Watson test. VIF was utilized to identify multicollinearity. Every outlier was removed.
→ The significance level was set at 0.05.

## 3. Results

In the analysis of gender-based mean differences in the scales and subscales SSES (socioemotional skills), perceived quality of life, life satisfaction, PYD (Positive Youth Development)|competence, confidence, and connection, and DASS (Depression, Anxiety, and Stress)|depression, anxiety and stress, statistically significant differences were found in the variables: SSES|optimism, emotional control, resilience/resistance, confidence, sociability, PYD = competence, confidence, and connection; DASS = depression, anxiety, and stress. No statistically significant differences existed between the dimensions of SSES|curiosity and persistence.

The male gender showed a higher mean across all domains except SSES|cooperation, relationship with teachers, and test anxiety, DASS|depression, anxiety, and stress (Table 1).

In the study of academic year differences, all variables (except DASS|anxiety) exhibit statistically significant differences when comparing differences in means.

Regarding lower secondary, the 7th grade has a higher average in the domains SSES|optimism, emotional control, resilience/resistance, confidence, curiosity, sociability, persistence/perseverance, creativity, energy, cooperation, self-control, sense of belonging to school, bullying, and relationships with teachers; PYD|competence, confidence, and connection; perceived quality of life and life satisfaction, in contrast to the 8th grade. The 8th grade has higher averages for DASS|stress and depression, with less favorable results.

With a more positive outlook, the 7th grade only stands out negatively in terms of bullying, while the 9th grade has a lower result.

However, in upper secondary, 10th graders have a higher average in the dimensions of optimism, emotional control, resilience/resistance, confidence, curiosity, sociability, persistence/perseverance, creativity, energy, cooperation, self-control, and a sense of belonging to school; PYD|competence, confidence, and connection; perceived quality of life and life satisfaction, compared to 12th graders. Year 12, excluding SSES|curiosity, persistence/perseverance, creativity, cooperation, self-control (11th grade), a sense of school belonging, and relationships with teachers. Year 11 has a higher mean for SSES | bullying and a lower mean for relationships with teachers than Year 12, which stands out favorably. In contrast to the 10th grade, the 12th grade has a higher average in the DASS|stress and depression.

In the 8th grade, the means of all SSES dimensions (except test anxiety), PYD, perceived quality of life, and life satisfaction decrease, except DASS|stress and depression, which tend to rise. The dimensions SSES, PYD, and perceived quality of life tend to increase in the 9th grade, then again in the 10th grade, before decreasing in the 11th grade, and, in some cases, tending to decrease in the year that follows (12th grade). Bullying and test anxiety tend to increase in 11th grade.

With an improved outlook in 7th grade, a decline in 8th grade, and an improvement in 9th grade, satisfaction with life tends to decline after 9th grade. DASS|stress and depression are also more positive in 7th grade, with an increase in 8th grade, an improvement in 9th grade, and successive increases in subsequent years (Table 2).

In the examination of variable correlations, a significant negative correlation was found between age and SSES|optimism, resilience/resistance, confidence, sociability, energy, sense of belonging to school, bullying, perceived quality of life, and life satisfaction; PYD|competence and connection, indicating that these domains tend to decline with age. Nonetheless, there is a significant positive correlation between age and SSES|cooperation, curiosity, and test anxiety, and DASS|depression and stress.

No significant correlations existed between age and the variables SSES|emotional control; PYD|confidence; DASS|anxiety.

Overall, significant positive correlations were found between all SSES subscales, suggesting that as one subscale increases, the others tend to increase as well, except bullying, which demonstrates a significant negative correlation with the other subscales. There is no significant correlation between test anxiety and curiosity and bullying, and there is a negative correlation between test anxiety and all subscales except cooperation and relationship with teachers.

All variables except SSES|bullying and test anxiety and DASS|depression, anxiety, and stress are significantly and positively correlated with perceived quality of life and life satisfaction. This analysis suggests that as life satisfaction and perceived quality of life increase, so do SSES capabilities.

PYD|competence correlates positively and significantly with all domains except age, bullying, test anxiety, and DASS dimensions. Except for the lack of a substantial correlation between age and PYD|confidence, the pattern holds for both PYD|confidence and connection.

DASS|stress has a significant correlation with all variables, both positively and negatively, with age, bullying, test anxiety, and other DASS domains|depression and stress. The DASS|depression and anxiety (correlation with age is not significant), exhibit the same trend (Table 3).

A multiple linear regression (Enter method) was conducted using the variable age, gender, PYD, SSES, and DASS to determine the predictability of perceived quality of life after checking for and deleting outliers. Multicollinearity was not an issue as the VIF never exceeded 10 in any of the variables, and the tolerance never fell below. The data do not satisfy the assumption of independent errors (Durbin–Watson value = 0.001). The histogram of standardized residuals and the normal P–P plot of standardized residuals indicated that the error distribution of the data was approximately normal. The data also support the hypothesis of non-zero variances.

**Figure 1:**
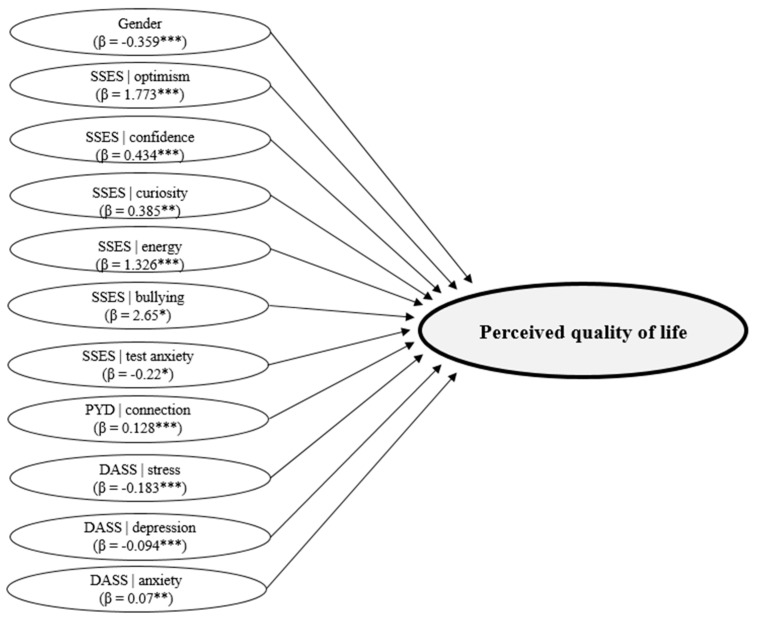
Variables that explain perceived quality of life. Note: *** *p* ˂ 0.001; ** *p* ˂ 0.01; * *p* ˂ 0.05.

In this study, a significant equation was observed (*F*(23.2804) = 186.812. *p* ˂ 0.001. R^2^ = 0.605. R^2^_Adjusted_ = 0.602), indicating that the independent variables explain 60.5% of the variance in perceived quality of life (Table 4). Figure 1 shows the variables with a significant impact on perceived quality of life.

Tests to determine whether the data satisfied the assumption of collinearity revealed that multicollinearity was not a concern as the VIF never exceeded 10 in any of the variables, and the tolerance never fell below 0.01. The data do not meet the assumption of independent errors (Durbin–Watson value = 0), pointing to positive autocorrelation. The histogram of standardized residuals and the normal P–P plot of standardized residuals indicated that the data contained approximately normally distributed errors. The data also met the assumption of non-zero variances.

**Figure 2:**
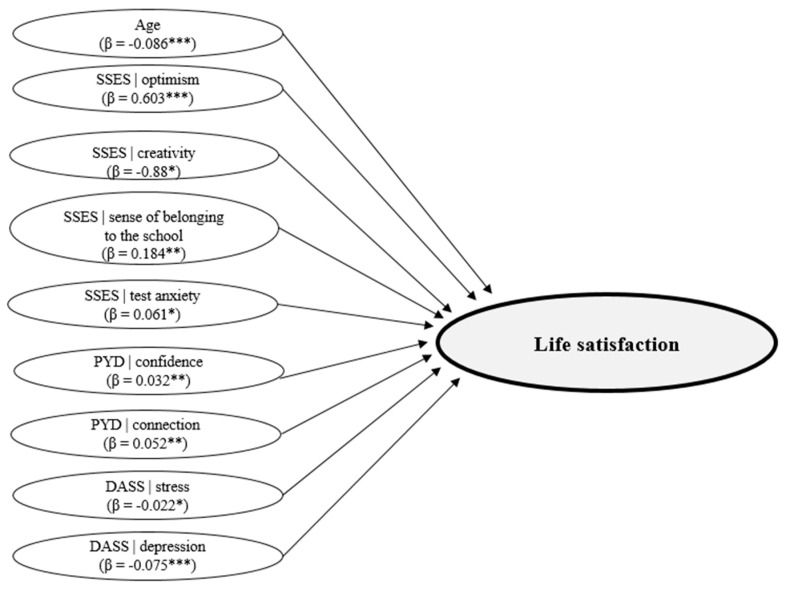
Variables that explain life satisfaction. Note: *** *p* ˂ 0.001; ** *p* ˂ 0.01; * *p* ˂ 0.05.

A multiple regression (Enter method) based on the previous set of independent variables but investigating their prediction of life satisfaction revealed a significant equation (*F*(23.2802) = 110.879. *p* ˂ 0.001. R^2^ = 0.69. R^2^_Adjusted_ = 0.47) revealing that the total variation in life satisfaction can be explained by 69% by age, gender, and the variables SSES, PYD, and DASS (Table 5). Figure 2 shows the variables with a significant impact on life satisfaction.

The analysis reveals only age, SSES|optimism, SSES creativity, SSES|sense of belonging to the school, SSES|test anxiety, SSES|stress, PYD|confidence, PYD|connection, DASS|stress, and DASS|depression significantly predict life satisfaction (Figure 2).

## 4. Discussion and Conclusions from the Perspective of a Young Person

The perception of quality of life among adolescents is not solely associated with individual competencies but rather with positive correlations between these competencies and the joint assessment of socioemotional well-being (SSES), positive youth development (PYD), and depression, anxiety, and stress symptoms (DASS). The results align with earlier studies that suggest that a person’s ability to adjust to environmental challenges has an impact on their overall well-being and quality of life [16,18,29]. Also, de la Barrera et al. [40] discovered that higher levels of socioemotional well-being contribute to greater life satisfaction. According to Lopes and Nihei [41], lower levels of well-being and diminished use of adaptive coping strategies are associated with lower life satisfaction. Similarly, psychological symptoms have been linked to decreased life satisfaction and health [42].

Gender differences exist for SSES, PYD, and DASS. Regarding gender, it was observed that SSES, DASS, PYD, perceived quality of life, and life satisfaction scores were lower for the female group in comparison to the male group. These findings are consistent with previous studies [20]. The lower self-esteem reported by the female group in previous research [25] was consistent with higher levels of school anxiety and DASS, and the greater ability for pro-social behavior [20] could explain a better teacher–student relationship in comparison with the male group. The male group stands out positively in most of the other SSES, revealing higher levels of self-management and motivation. According to previous literature, females were more likely to experience psychological distress symptoms, namely anxiety, stress, and depression, particularly during the pandemic [27,43]. These results can be explained by the previous findings of Gomez-Baya et al. [25], who discovered that females have lower self-esteem, self-concept, and well-being, resulting in negative self-perceptions. In contrast, males tend to have higher levels of social adjustment, which enables them to better adapt to daily challenges and enhance their quality of life, well-being, and overall satisfaction. The fact that the pandemic has had a greater negative impact on women may contribute to or enhance this phenomenon [16,17,18].

SSES, PYD, and DASS vary with age. Aging (measured by the school year proxy) was associated with lower levels of emotional health, optimism, resiliency, self-confidence, and sociability. Prior studies have shown a connection between advancing age, increased socioemotional skills deficits, and psychological symptoms [40], which lends support to this phenomenon.

As youth mature and approach maturity, the indicator of bullying tends to decrease. Consistent with the present study, the HBSC 2022 study in Portugal found that younger people were more likely to report being abusers or victims of bullying [44]. Similarly, a study conducted in the United States found that bullying decreased as individuals aged [45]. Bullying continues to be one of the age group’s greatest scourges [2,8]. Although it decreases with age and education, it hinders an increase in life satisfaction and correlates positively with the emergence of psychological symptoms [46].

In our models, aging (measured by the school year proxy) was associated with a lower perception of life satisfaction, but not with perceived quality of life. Growing up implies maturation, but the results indicate a negative impact on the young person’s socio-emotional state. Literature has shown that as individuals grow and develop, they are faced with increasingly complex and difficult challenges related to their personal and environmental circumstances. This can include academic pressure and family issues [47,48]. Making less healthy adjustments to environmental challenges can lead to a decrease in emotional health, well-being, and overall quality of life [16,18,29]. This may be a plausible explanation for the negative influence of DASS|depression and stress on life satisfaction and DASS|depression, anxiety, and stress on quality and perceived life.

In adolescents’ own words, when students are required to take entrance examinations for higher education in the twelfth grade, test anxiety tends to increase relative to other grades, possibly due to the increased importance of the exams. The difficulties associated with rising student development and school demands may help to explain the negative correlation between this variable and the majority of socioemotional skills. Similar results were found in a national study by Matos et al. [49] analyzing the individual and contextual factors of adolescents’ well-being and life satisfaction during COVID-19. The majority of adolescents who perceived their life at school to be significantly worse had high levels of psychological symptomatology and low levels of life satisfaction. The psychological health and well-being of Portuguese students appeared to decline with age and throughout their academic careers, indicating a concerning trend. Social restrictions and family issues, exacerbated by the pandemic, acted as risk factors for this phenomenon [1,15].

Age is not a predictor of quality of life perception, unlike gender, SSES, PYD, and DASS. Age, SSES, PYD, and DASS can all predict life satisfaction, but gender is not one of them. According to our results, satisfaction with life is also impacted by SSES|optimism, sense of belonging to school, PYD|confidence, and connection. For their part, SSES|optimism, confidence, curiosity, energy, and PYD|connection reveal a favorable impact on the explanation of perceived quality of life. An interesting result from our perspective is the fact that SSES|bullying does not reveal a negative relationship with perceived quality of life, and in the literature, bullying shows a negative impact on health-related quality of life [49], or the SSES | test anxiety did not reveal a decrease in the life satisfaction variable. as was observed with perceived quality of life. Since both models have an R-squared greater than 0.51, although less than 0.99, they are considered acceptable and have mild strength. In our research, we did not find literature supporting these findings.

It is crucial to understand that, in addition to their academic environment, adolescents’ familial environment has a significant impact on their mental health. According to Matos et al.’s study [50], adolescents who reported negative perceptions of school, Family, friends, themselves, and life had significantly more symptoms and less life satisfaction. Based on this research, we can assert that if we want to improve the psychological health and well-being of adolescents, and consequently their quality and satisfaction with life, we must:

- Promote socio-emotional skills and literacy, adapting to the gender and years of schooling specifics identified here, e.g., proactive work;
- Increase (or acquisition) clinical psychologists in schools and health facilities, e.g., preventive work;
- Avoid reactive work, which is required in instances of moderate or severe symptomatology or pathology.

### 4.1. Strengths and Limitations

Regarding the study’s strengths, it is important to note that it was conducted during the first school year after the return to the new post-COVID-19 normality, allowing us to see the actual situation and the psychological health requirements and characteristics of Portuguese students. In addition, the size of the sample and its stratification by the Portuguese mainland territories enable a country-wide perspective. Third, a period of development analysis between lower and upper secondary has different features. On the other hand, we note that the study is cross-sectional, we cannot be sure that the sample is representative, and we only used one method to collect data.

### 4.2. Future Research Directions

In a subsequent study, in order to comprehend how the model behaves, a more in-depth examination will be conducted by education level and gender.

## Figures and Tables

**Table 1:** ANOVA comparisons according to gender.

	Gender	n	*M*	*SD*	*F*
SSES|optimism	M	1460	2.782	0.763	227.562 ***
	F	1600	2.363	0.771	
SSES|emotional control	M	1450	2.400	0.714	354.826 ***
	F	1594	1.904	0.738	
SSES|resilience/resistance	M	1450	2.321	0.795	655.466 ***
	F	1594	1.577	0.806	
SSES|confidence	M	1448	2.265	0.726	102.895 ***
	F	1590	1.998	0.725	
SSES|curiosity	M	1444	2.689	0.672	2.642
	F	1582	2.727	0.617	
SSES|sociability	M	1438	2.555	0.708	91.011 ***
	F	1585	2.302	0.747	
SSES|persistence/perseverance	M	1446	2.643	0.672	3.109
	F	1584	2.599	0.703	
SSES|creativity	M	1438	2.601	0.658	17.490 ***
	F	1580	2.501	0.651	
SSES|energy	M	1433	2.532	0.713	245.290 ***
	F	1583	2.132	0.691	
SSES|cooperation	M	1429	2.932	0.599	50.007 ***
	F	1582	3.083	0.568	
SSES|self-control	M	1428	2.573	0.634	3.881 *
	F	1576	2.526	0.661	
SSES|sense of belonging to the school	M	1417	2.563	0.524	96.079 ***
	F	1572	2.371	0.545	
SSES|bullying	M	1409	0.323	0.557	14.656 ***
	F	1568	0.250	0.483	
SSES|relationships with teachers	M	1408	2.310	0.798	5.622 *
	F	1566	2.377	0.749	
SSES|test anxiety	M	1409	2.268	1.009	383.207 ***
	F	1563	2.966	0.934	
Perceived quality of life	M	1483	16.078	4.951	316.336 ***
	F	1615	12.835	5.176	
Life satisfaction	M	1481	7.377	1.809	92.491 ***
	F	1615	6.746	1.842	
PYD|competence	M	1403	15.046	4.350	302.636 ***
	F	1554	12.194	4.541	
PYD|confidence	M	1414	15.950	5.064	231.207 ***
	F	1571	12.942	5.678	
PYD|connection	M	1411	20.871	5.741	44.135 ***
	F	1568	19.471	5.748	
DASS|stress	M	1455	4.185	4.168	307.136 ***
	F	1588	7.073	4.858	
DASS|depression	M	1453	3.755	4.251	203.080 ***
	F	1588	6.196	5.108	
DASS|anxiety	M	1453	2.862	3.451	285.947 ***
	F	1588	5.437	4.774	

Note: *** *p* ˂ 0.001; * *p* ˂ 0.05.

**Table 2:** ANOVA comparisons according to year of schooling.

	School Year	*n*	*M*	*SD*	*F*
SSES|optimism	7th grade	463	2.75	0.81	13.791 ***
	8th grade	529	2.54	0.80	
	9th grade	521	2.60	0.82	
	10th grade	444	2.62	0.81	
	11th grade	739	2.45	0.77	
	12th grade	485	2.39	0.77	
SSES|emotional control	7th grade	458	2.22	0.78	3.833 **
	8th grade	526	2.04	0.76	
	9th grade	519	2.13	0.78	
	10th grade	442	2.19	0.76	
	11th grade	734	2.15	0.75	
	12th grade	485	2.07	0.80	
SSES|resilience/resistance	7th grade	455	2.14	0.90	8.511 ***
	8th grade	525	1.94	0.85	
	9th grade	521	1.94	0.91	
	10th grade	442	1.94	0.90	
	11th grade	735	1.86	0.89	
	12th grade	486	1.79	0.83	
SSES|confidence	7th grade	458	2.28	0.80	6.029 ***
	8th grade	525	2.08	0.75	
	9th grade	517	2.09	0.75	
	10th grade	443	2.15	0.74	
	11th grade	734	2.07	0.72	
	12th grade	482	2.06	0.70	
SSES|curiosity	7th grade	458	2.73	0.67	9.341 ***
	8th grade	524	2.55	0.69	
	9th grade	509	2.68	0.64	
	10th grade	442	2.82	0.64	
	11th grade	733	2.71	0.62	
	12th grade	482	2.74	0.60	
SSES|sociability	7th grade	458	2.59	0.77	10.332 ***
	8th grade	521	2.40	0.76	
	9th grade	512	2.45	0.75	
	10th grade	443	2.46	0.74	
	11th grade	729	2.33	0.74	
	12th grade	481	2.29	0.69	
SSES|persistence/perseverance	7th grade	458	2.66	0.71	4.205 **
	8th grade	524	2.50	0.71	
	9th grade	513	2.61	0.71	
	10th grade	442	2.68	0.67	
	11th grade	729	2.60	0.67	
	12th grade	485	2.62	0.70	
SSES|creativity	7th grade	459	2.63	0.66	5.947 ***
	8th grade	516	2.45	0.66	
	9th grade	513	2.53	0.67	
	10th grade	437	2.65	0.62	
	11th grade	732	2.52	0.66	
	12th grade	482	2.55	0.65	
SSES|energy	7th grade	457	2.49	0.74	11.491 ***
	8th grade	519	2.28	0.71	
	9th grade	508	2.33	0.76	
	10th grade	438	2.40	0.74	
	11th grade	731	2.22	0.73	
	12th grade	483	2.20	0.73	
SSES|cooperation	7th grade	456	3.02	0.62	4.590 ***
	8th grade	512	2.91	0.62	
	9th grade	511	2.98	0.60	
	10th grade	442	3.09	0.56	
	11th grade	726	3.00	0.58	
	12th grade	483	3.03	0.58	
SSES|self-control	7th grade	453	2.60	0.65	4.169 **
	8th grade	514	2.45	0.64	
	9th grade	508	2.53	0.67	
	10th grade	439	2.62	0.65	
	11th grade	729	2.53	0.63	
	12th grade	480	2.53	0.70	
SSES|sense of belonging to the school	7th grade	456	2.55	0.57	6.466 ***
	8th grade	503	2.40	0.53	
	9th grade	505	2.46	0.56	
	10th grade	435	2.52	0.54	
	11th grade	725	2.41	0.53	
	12th grade	479	2.43	0.55	
SSES|bullying	7th grade	452	0.46	0.67	20.581 ***
	8th grade	501	0.38	0.59	
	9th grade	502	0.31	0.57	
	10th grade	435	0.22	0.44	
	11th grade	722	0.23	0.42	
	12th grade	477	0.18	0.41	
SSES|relationships with teachers	7th grade	455	2.42	0.75	7.470 ***
	8th grade	497	2.19	0.85	
	9th grade	501	2.29	0.81	
	10th grade	434	2.42	0.73	
	11th grade	723	2.32	0.76	
	12th grade	478	2.44	0.69	
SSES|test anxiety	7th grade	451	2.43	0.99	4.481 ***
	8th grade	501	2.67	0.98	
	9th grade	500	2.59	1.07	
	10th grade	432	2.67	1.08	
	11th grade	721	2.70	1.03	
	12th grade	478	2.68	1.06	
Perceived quality of life	7th grade	476	16.44	5.19	27.289 ***
	8th grade	540	14.38	5.56	
	9th grade	525	14.46	5.51	
	10th grade	447	14.60	5.30	
	11th grade	744	13.49	5.12	
	12th grade	491	12.84	4.88	
Life satisfaction	7th grade	475	7.64	1.87	18.020 ***
	8th grade	541	7.03	1.89	
	9th grade	526	7.10	1.72	
	10th grade	446	7.07	1.88	
	11th grade	742	6.75	1.83	
	12th grade	491	6.65	1.89	
PYD|competence	7th grade	455	14.66	4.87	10.810 ***
	8th grade	504	13.29	4.90	
	9th grade	505	13.59	4.68	
	10th grade	422	13.88	4.75	
	11th grade	707	12.94	4.53	
	12th grade	480	12.74	4.50	
PYD|confidence	7th grade	458	15.36	5.78	8.237 ***
	8th grade	512	13.66	5.83	
	9th grade	512	14.35	5.74	
	10th grade	427	15.02	5.66	
	11th grade	711	13.86	5.41	
	12th grade	481	13.62	5.45	
PYD|connection	7th grade	458	21.80	6.35	15.920 ***
	8th grade	512	19.63	5.97	
	9th grade	511	20.17	5.81	
	10th grade	424	20.83	5.61	
	11th grade	710	19.29	5.54	
	12th grade	480	19.06	5.29	
DASS|stress	7th grade	472	4.91	4.58	7.484 ***
	8th grade	537	5.69	4.79	
	9th grade	524	5.38	4.62	
	10th grade	429	5.84	4.85	
	11th grade	715	6.02	4.87	
	12th grade	487	6.64	4.93	
DASS|depression	7th grade	471	4.21	4.65	6.686 ***
	8th grade	536	5.31	5.06	
	9th grade	524	4.76	4.72	
	10th grade	429	5.18	5.08	
	11th grade	715	5.36	4.80	
	12th grade	487	5.89	5.25	
DASS|anxiety	7th grade	471	3.96	4.43	2.077
	8th grade	536	4.72	4.68	
	9th grade	524	4.15	4.37	
	10th grade	429	4.05	4.45	
	11th grade	715	4.25	4.28	
	12th grade	487	4.48	4.50	

Note: *** *p* ˂ 0.001; ** *p* ˂ 0.01.

**Table 3:** Correlation between variables.

	1	2	3	4	5	6	7	8	9	10	11	12	13	14	15	16	17	18	19	20	21	22	23	24
1. Age	1																							
2. SSES|optimism	−0.102 ***																							
3. SSES|emotional control	0.028	0.577 ***																						
4. SSES|resilience/resistance	−0.077 ***	0.553 ***	0.652 ***																					
5. SSES|confidence	−0.076 ***	0.555 ***	0.436 ***	0.348 ***																				
6. SSES|curiosity	0.046 *	0.381 ***	0.235 ***	0.083 ***	0.291 ***																			
7. SSES|sociability	−0.105 ***	0.553 ***	0.345 ***	0.351 ***	0.519 ***	0.322 ***																		
8. SSES|persistence/perseverance	0.008	0.409 ***	0.351 ***	0.230 ***	0.287 ***	0.503 ***	0.323 ***																	
9. SSES|creativity	0.000	0.309 ***	0.207 ***	0.179 ***	0.167 ***	0.444 ***	0.326 ***	0.393 ***																
10. SSES|energy	−0.093 ***	0.625 ***	0.445 ***	0.453 ***	0.436 ***	0.393 ***	0.618 ***	0.460 ***	0.395 ***															
11. SSES|cooperation	0.067 ***	0.335 ***	0.258 ***	0.049 **	0.363 ***	0.487 ***	0.412 ***	0.472 ***	0.343 ***	0.371 ***														
12. SSES|self-control	0.034	0.333 ***	0.434 ***	0.215 ***	0.296 ***	0.406 ***	0.189 ***	0.491 ***	0.313 ***	0.298 ***	0.509 ***													
13. SSES|sense of belonging to school	−0.050 **	0.570 ***	0.424 ***	0.411 ***	0.494 ***	0.305 ***	0.650 ***	0.364 ***	0.291 ***	0.550 ***	0.403 ***	0.271 ***												
14. SSES|bullying	−0.174 ***	−0.196 ***	−0.205 ***	−0.140 ***	−0.231 ***	−0.168 ***	−0.152 ***	−0.179 ***	−0.069 ***	−0.112 ***	−0.230 ***	−0.185 ***	−0.272 ***											
15. SSES|relationships with teachers	0.002	0.196 ***	0.129 ***	0.054 **	0.241 ***	0.285 ***	0.141 ***	0.232 ***	0.143 ***	0.152 ***	0.297 ***	0.232 ***	0.234 ***	−0.137 ***										
16. SSES|test anxiety	0.038 *	−0.254 ***	−0.342 ***	−0.533 ***	−0.149 ***	0.019	−0.155 ***	−0.086 ***	−0.110 ***	−0.229 ***	0.123 ***	−0.043 *	−0.165 ***	0.014	0.107 ***									
17. Life satisfaction	−0.155 ***	0.597 ***	0.397 ***	0.397 ***	0.378 ***	0.235 ***	0.372 ***	0.290 ***	0.175 ***	0.431 ***	0.225 ***	0.236 ***	0.439 ***	−0.166 ***	0.192 ***	−0.169 ***								
18. Perceived quality of life	−0.179 ***	0.677 ***	0.496 ***	0.505 ***	0.482 ***	0.277 ***	0.464 ***	0.324 ***	0.224 ***	0.581 ***	0.229 ***	0.253 ***	0.467 ***	−0.123 ***	0.169 ***	−0.282 ***	0.585 ***							
19. PYD|competence	−0.084 ***	0.597 ***	0.423 ***	0.445 ***	0.448 ***	0.349 ***	0.633 ***	0.451 ***	0.377 ***	0.667 ***	0.345 ***	0.324 ***	0.602 ***	−0.147 ***	0.170 ***	−0.257 ***	0.438 ***	0.518 ***						
20. PYD|confidence	−0.025	0.712 ***	0.505 ***	0.509 ***	0.447 ***	0.336 ***	0.505 ***	0.404 ***	0.338 ***	0.595 ***	0.318 ***	0.342 ***	0.537 ***	−0.175 ***	0.183 ***	−0.279 ***	0.516 ***	0.565 ***	0.704 ***					
21. PYD|connection	−0.101 ***	0.633 ***	0.423 ***	0.359 ***	0.599 ***	0.410 ***	0.526 ***	0.416 ***	0.285 ***	0.532 ***	0.457 ***	0.358 ***	0.569 ***	−0.209 ***	0.362 ***	−0.114 ***	0.519 ***	0.551 ***	0.596 ***	0.631 ***				
22.DASS-21|stress	0.076 ***	−0.603 ***	−0.616 ***	−0.623 ***	−0.421 ***	−0.117 ***	−0.357 ***	−0.245 ***	−0.131 ***	−0.422 ***	−0.136 ***	−0.261 ***	−0.419 ***	0.212 ***	−0.112 ***	0.350 ***	−0.478 ***	−0.566 ***	−0.421 ***	−0.512 ***	−0.444 ***			
23.DASS-21|depression	0.073 ***	−0.724 ***	−0.524 ***	−0.542 ***	−0.449 ***	−0.241 ***	−0.448 ***	−0.360 ***	−0.202 ***	−0.517 ***	−0.176 ***	−0.260 ***	−0.510 ***	0.237 ***	−0.156 ***	0.274 ***	−0.572 ***	−0.608 ***	−0.506 ***	−0.615 ***	−0.522 ***	0.758 ***		
24.DASS-21|anxiety	−0.005	−0.564 ***	−0.536 ***	−0.579 ***	−0.379 ***	−0.154 ***	−0.349 ***	−0.274 ***	−0.147 ***	−0.433 ***	−0.138 ***	−0.225 ***	−0.432 ***	0.243 ***	−0.137 ***	0.323 ***	−0.436 ***	−0.494 ***	−0.429 ***	−0.505 ***	−0.412 ***	0.795 ***	0.738 ***	

Notes: *** *p* ˂ 0.001; ** *p* ˂ 0.01; * *p* ˂ 0.05. The variables on the y-axis are numbered. The same being assumed on the x-axis.

**Table 4:** Multiple Linear regression for perceived quality of life (Enter method).

	*B*	Error	*Beta*	*t*	*Collinearity Statistics*	
(Constant)	8.484	0.833		***	***Tolerance***	***VIF***
Age	−0.239	0.036	−0.084	−6.686	0.89	1.123
Gender	**−0.359**	0.108	−0.043	−3.313 ***	0.832	1.202
SSES|optimism	**1.773**	0.149	0.269	11.92 ***	0.276	3.618
SSES|emotional control	0.244	0.127	0.036	1.92	0.404	2.477
SSES|resilience/resistance	0.139	0.118	0.023	1.181	0.367	2.728
SSES|confidence	**0.434**	0.117	0.061	3.714 ***	0.531	1.885
SSES|curiosity	**0.385**	0.13	0.046	2.958 **	0.578	1.73
SSES|sociability	−0.118	0.134	−0.017	−0.884	0.395	2.529
SSES|persistence/perseverance	−0.148	0.122	−0.019	−1.208	0.548	1.825
SSES|creativity	−0.181	0.114	−0.023	−1.588	0.69	1.449
SSES|energy	**1.326**	0.134	0.186	9.997 ***	0.4	2.502
SSES|cooperation	−0.168	0.154	−0.018	−1.091	0.492	2.031
SSES|self-control	−0.184	0.127	−0.023	−1.443	0.56	1.786
SSES|sense of belonging to the school	−0.11	0.175	−0.011	−0.63	0.425	2.352
SSES|bullying	**0.265**	0.134	0.026	1.977 *	0.822	1.217
SSES | relationships with teachers	0.156	0.092	0.022	1.703	0.808	1.238
SSES|test anxiety	**−0** **.220**	0.075	−0.043	−2.929 **	0.66	1.515
PYD|competence	0.019	0.023	0.016	0.8	0.335	2.985
PYD|confidence	0.025	0.019	0.027	1.298	0.33	3.026
PYD|connection	**0.128**	0.018	0.137	7.097 ***	0.376	2.658
DASS|stress	**−0.183**	0.026	−0.165	−7.103 ***	0.26	3.843
DASS|depression	**−0.094**	−0.025	−0.088	−3.818 ***	0.266	3.753
DASS|anxiety	**0.07**	0.025	0.058	2.753 **	0.315	3.174

Note: *** *p* ˂ 0.001; ** *p* ˂ 0.01; * *p* ˂ 0.05. Bold = significant differences.

**Table 5:** Multiple linear regression for life satisfaction (Enter method).

	*B*	Error	*Beta*	*t*	*Collinearity Statistics*	
(Constant)	5.282	0.325		16.239 ***	***Tolerance***	***VIF***
Age	**−0.086**	0.014	−0.089	−6.139 ***	0.893	1.12
Gender	0.008	0.042	0.003	0.856	0.831	1.203
SSES|optimism	**0.603**	0.058	0.27	10.443 ***	0.279	3.583
SSES|emotional control	−0.034	0.05	−0.015	−0.686	0.404	2.476
SSES|resilience/resistance	0.066	0.046	0.032	1.434	0.37	2.703
SSES|confidence	−0.064	0.046	−0.026	−1.404	0.526	1.901
SSES|curiosity	0.024	0.051	0.009	0.479	0.576	1.737
SSES|sociability	−0.065	0.052	−0.027	−1.243	0.393	2.544
SSES|persistence/perseverance	−0.031	0.048	−0.012	−0.657	0.551	1.816
SSES|creativity	**−0.88**	0.045	−0.032	−1.964 *	0.692	1.445
SSES|energy	0.055	0.053	0.023	1.039	0.397	2.518
SSES|cooperation	−0.036	0.06	−0.012	−0.596	0.491	2.036
SSES|self-control	0.028	0.05	0.01	0.567	0.56	1.785
SSES|sense of belonging to the school	**0.184**	0.069	0.056	2.681 **	0.424	2.358
SSES|bullying	0.001	0.053	0.000	0.013	0.821	1.217
SSES|relationships with teachers	0.048	0.036	0.021	1.349	0.806	1.241
SSES|test anxiety	**0.061**	0.029	0.035	2.073 *	0.66	1.515
PYD|competence	0.003	0.009	0.007	0.3	0.334	2.991
PYD|confidence	**0.032**	0.008	0.101	4.235 **	0.331	3.024
PYD|connection	**0.052**	0.007	0.163	7.32 **	0.375	2.667
DASS|stress	**−0.022**	0.01	−0.057	−2.143 *	0.26	3.84
DASS|depression	**−0.075**	0.01	−0.204	−7.705 ***	0.267	3.749
DASS|anxiety	0.016	0.01	0.039	1.595	0.315	3.175

Note: *** *p* ˂ 0.001; ** *p* ˂ 0.01; * *p* ˂ 0.05. Bold = significant differences.

## Data Availability

https://www.dgeec.mec.pt/np4/1357.html, accessed on 5 July 2023.
